# Emerging trends in sperm selection: enhancing success rates in assisted reproduction

**DOI:** 10.1186/s12958-024-01239-1

**Published:** 2024-06-14

**Authors:** Xiang Zhang, Shuen Chao, Ningxin Ye, Dongfang Ouyang

**Affiliations:** 1 College of Animal Science and Technology, China Agricultural University, Beijing, 100193 China; 2 Center for Engineering in Medicine and Surgery, Massachusetts General Hospital, Harvard Medical School, Charlestown, MA 02129 USA; 3 Department of Mechanical and Industrial Engineering, University of Toronto, Toronto, M5S 3G8 Canada; 4 Department of Biology, University of Ottawa, Ottawa, ON K1N 6N5 Canada

**Keywords:** Assisted Reproductive Technology (ART), Sperm selection techniques, Intracytoplasmic sperm injection (ICSI), Sperm Quality Assessment

## Abstract

This comprehensive review explores the evolving landscape of sperm selection techniques within the realm of Assisted Reproductive Technology (ART). Our analysis delves into a range of methods from traditional approaches like density gradient centrifugation to advanced techniques such as Magnetic-Activated Cell Sorting (MACS) and Intracytoplasmic Morphologically Selected Sperm Injection (IMSI). We critically assess the efficacy of these methods in terms of sperm motility, morphology, DNA integrity, and other functional attributes, providing a detailed comparison of their clinical outcomes. We highlight the transition from conventional sperm selection methods, which primarily focus on physical characteristics, to more sophisticated techniques that offer a comprehensive evaluation of sperm molecular properties. This shift not only promises enhanced prediction of fertilization success but also has significant implications for improving embryo quality and increasing the chances of live birth. By synthesizing various studies and research papers, we present an in-depth analysis of the predictability of different sperm selection procedures in ART. The review also discusses the clinical applicability of these methods, emphasizing their potential in shaping the future of assisted reproduction. Our findings suggest that the integration of advanced sperm selection strategies in ART could lead to more cost-effective treatments with reduced duration and higher success rates. This review aims to provide clinicians and researchers in reproductive medicine with comprehensive insights into the current state and future prospects of sperm selection technologies in ART.

## Background

The field of Assisted Reproductive Technology (ART) has experienced significant transformation with the advent of advanced sperm selection techniques [1–3]. This section thoroughly explores various innovative methods crucial in gamete and embryo selection, substantially advancing fertility treatments [4–8]. Techniques such as microfluidic sperm sorting, Magnetic-Activated Cell Sorting (MACS) [6, 8, 9], electrophoretic sperm selection, Intracytoplasmic Morphologically Selected Sperm Injection (IMSI) [10–12], and sperm DNA fragmentation analysis [13–15] are pivotal in reshaping the ART landscape. They address essential aspects of gamete quality evaluation and selection [4, 6, 10, 14, 15]. These subsections offer detailed insights into the benefits, limitations, and developmental trajectory of each method, clarifying their roles in improving reproductive outcomes [8, 10, 13–15]. Additionally, a comparative analysis provides a comprehensive view of traditional and advanced sperm selection methods, underscoring the transformative impact of these innovative approaches [4, 7, 8, 10, 14]. Moreover, this section delineates future directions and implications, spotlighting ongoing advancements poised to revolutionize ART, with a focus on more personalized, accessible, and ethically responsible fertility treatments [3, 10, 14–16].

## Advanced sperm selection techniques

### Microfluidic sperm sorting

**Fig. 1:**
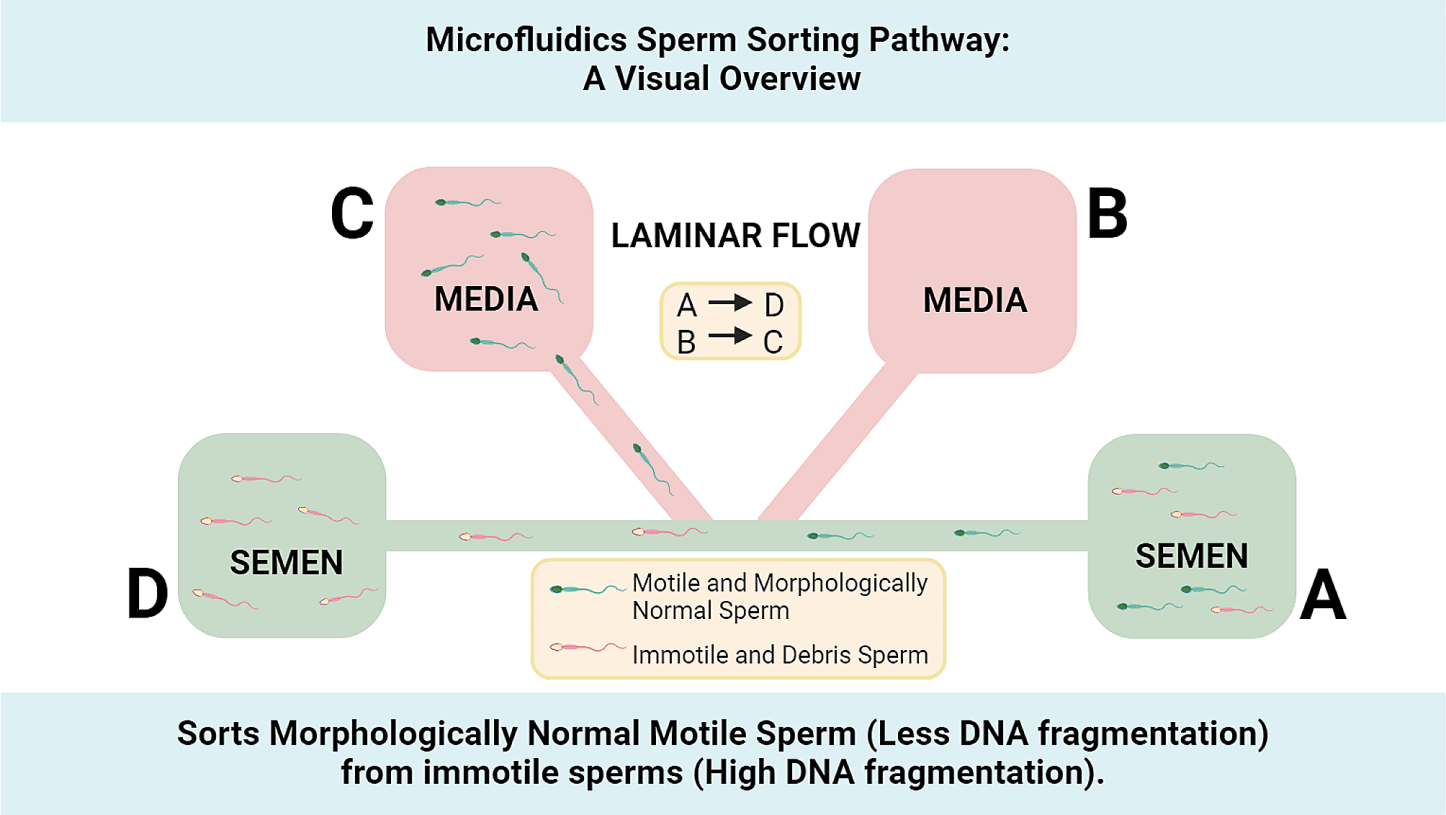
Microfluidics Sperm Sorting Pathway: A Visual Overview

Microfluidic sperm selection techniques have emerged as advanced methods for isolating and sorting motile spermatozoa based on their functionality and morphology. These techniques utilize microfluidic devices that are fabricated using materials such as polydimethylsiloxane (PDMS), a silicon-based organic polymer. The devices consist of microchannels with specific dimensions tailored to the size of sperm cells. Creating flow conditions within the microchannels allows for the separation and collection of motile and morphologically normal spermatozoa, while non-motile spermatozoa and debris exit through a separate outlet(Fig. 1) [1–3, 17].

During the sorting process of sperm selection through microfluidic techniques, semen samples are introduced into the microfluidic devices. To isolate motile spermatozoa, different strategies are employed depending on the device design. These strategies may involve the use of parallel streams with varying widths, allowing motile spermatozoa to deviate into one stream while non-motile spermatozoa and debris continue along their initial streamlines. Other devices may utilize microporous membranes or precise flow control to separate motile spermatozoa from other components of the semen sample.

Microfluidic sperm selection techniques offer several advantages over conventional methods such as density gradient centrifugation and swim-up techniques (Table 1). These microfluidic techniques provide high selectivity and specificity in isolating motile and morphologically normal spermatozoa, resulting in improved sperm selection processes. Additionally, microfluidic devices enable real-time monitoring and analysis of sperm cells, allowing for precise selection based on parameters such as motility, morphology, and DNA integrity. The small-scale nature of microfluidic devices also allows for reduced sample volumes which improv handling of individual sperm cells.

However, it is important to note that microfluidic sperm selection techniques also possess limitations, including manipulation requirements, the potential of device clogging, and the cost of the device. One limitation is the complexity of device fabrication and the requirement for specialized equipment and expertise. The fabrication process involves precise control over microchannel dimensions and the integration of microfluidic components, thereby posing challenges in terms of scalability and accessibility. Another limitation is the potential for device clogging or blockage due to the presence of debris or non-motile spermatozoa in the semen sample. While microfluidic devices aim to separate motile spermatozoa from other components, the presence of debris or non-motile spermatozoa can adversely affect the sorting efficiency and accuracy of the technique. Furthermore, the cost associated with microfluidic sperm selection techniques may be higher compared to conventional methods. The fabrication of microfluidic devices along with the requirement for specialized equipment and materials contribute to the overall cost, which may limit their widespread adoption in certain settings or regions with limited resources.

In summary, microfluidic sperm selection techniques offer advanced capabilities for sperm selection in assisted reproductive technology. These techniques afford high selectivity and real-time analysis of sperm parameters, thereby leading to the enhanced quality of sperm. However, limitations such as the complexity of device fabrication, potential for device clogging, and high cost should be considered. The intricacy involved in the fabrication of devices for microfluidic sperm selection arises from the need for precise engineering and manufacturing processes. Fabricating microchannels with specific dimensions and integrating microfluidic components necessitate specialized expertise and equipment, often requiring cleanroom facilities. The intricate design and assembly process can be time-consuming and challenging, which may hinder widespread adoption, particularly in settings with limited resources where access to advanced fabrication techniques is limited.

One of the critical limitations of microfluidic sperm selection techniques is the potential for device clogging or blockage during the processing of semen samples. The microfluidic devices depend on the controlled fluid flow through microchannels to sort and isolate spermatozoa based on their motility and morphology. However, the presence of debris, non-motile spermatozoa, or other particulate matter in the semen sample may obstruct the microchannels, thereby compromising the accuracy and efficiency of the sperm sorting process. To mitigate this issue, researchers have been actively working on innovative solutions to prevent device clogging and enhance the performance of microfluidic platforms. For instance, Venugopal et al. [18] introduced a microfluidic platform with an array of uniquely designed multifunctional microposts to achieve higher capture efficiency and flow rates, while effectively avoiding clogging issues. They employed an alternative carry-forward path that allowed particles to bypass congested areas, mitigating the detrimental effects of surge pressure build-up and shear stress on cell viability.

Another approach to addressing clogging concerns is the bioinspired lobe filter system developed by Clark and San-Miguel [19]. Inspired by the filtration mechanism of manta rays, their microfluidic lobe filters enable efficient filtration of particles in the range of 10–30 μm with precise control and high throughput. The filtration efficiency increased with fluid flow rate, thereby highlighting the role of particle inertial effects in lobe filter separation. These innovations promise to significantly improve the reliability and performance of microfluidic sperm selection techniques, making them more suitable for high-throughput and continuous applications in assisted reproductive technology. In summary, while clogging remains a limitation in microfluidic sperm selection, ongoing research and the development of novel microfluidic designs, such as those inspired by nature and incorporating alternative carry-forward paths, demonstrate significant potential in overcoming this challenge and enhancing the effectiveness of microfluidic sperm sorting for ART.

The exorbitant price associated with microfluidic sperm selection techniques can pose financial challenges for healthcare facilities and patients. The fabrication of microfluidic devices requires specialized materials and equipment, thus contributing to their initial expenses. Additionally, the need for skilled personnel and proper training escalates the overall cost. While the potential benefits of improved sperm quality and ART outcomes are significant, cost considerations may hinder the widespread adoption of these techniques, particularly in regions with limited resources.

To surmount these limitations and fully connect the potential of microfluidic sperm selection techniques in assisted reproductive technology, a synergistic approach involving researchers, clinicians, and industry partners is imperative. Such collaborative endeavors can drive progress in multiple key domains, enhancing the accessibility, efficacy, and cost-effectiveness of these nascent technologies. Researchers could concentrate on simplifying device fabrication processes and refining device designs to mitigate the complexity and cost concerns. Additionally, explorations into novel sample preparation methods can ameliorate the risk of device clogging, ensuring more reliable and consistent results. Clinicians occupy a pivotal role in orchestrating extensive clinical trials to validate the long-term efficacy and impact of microfluidic sperm selection techniques on ART success rates. Collaboration with industry partners, strides in manufacturing technologies and materials can be actualized, thereby potentially diminishing the overall financial burden associated with microfluidic devices and facilitating their affordability for a broader range of healthcare facilities and patients. Through such collaborative endeavors, the field of assisted reproductive technology stands to make substantial progress, thereby extending personalized and effective fertility treatments to couples on a global scale.

### Magnetic-activated cell sorting (MACS)

**Fig. 2:**
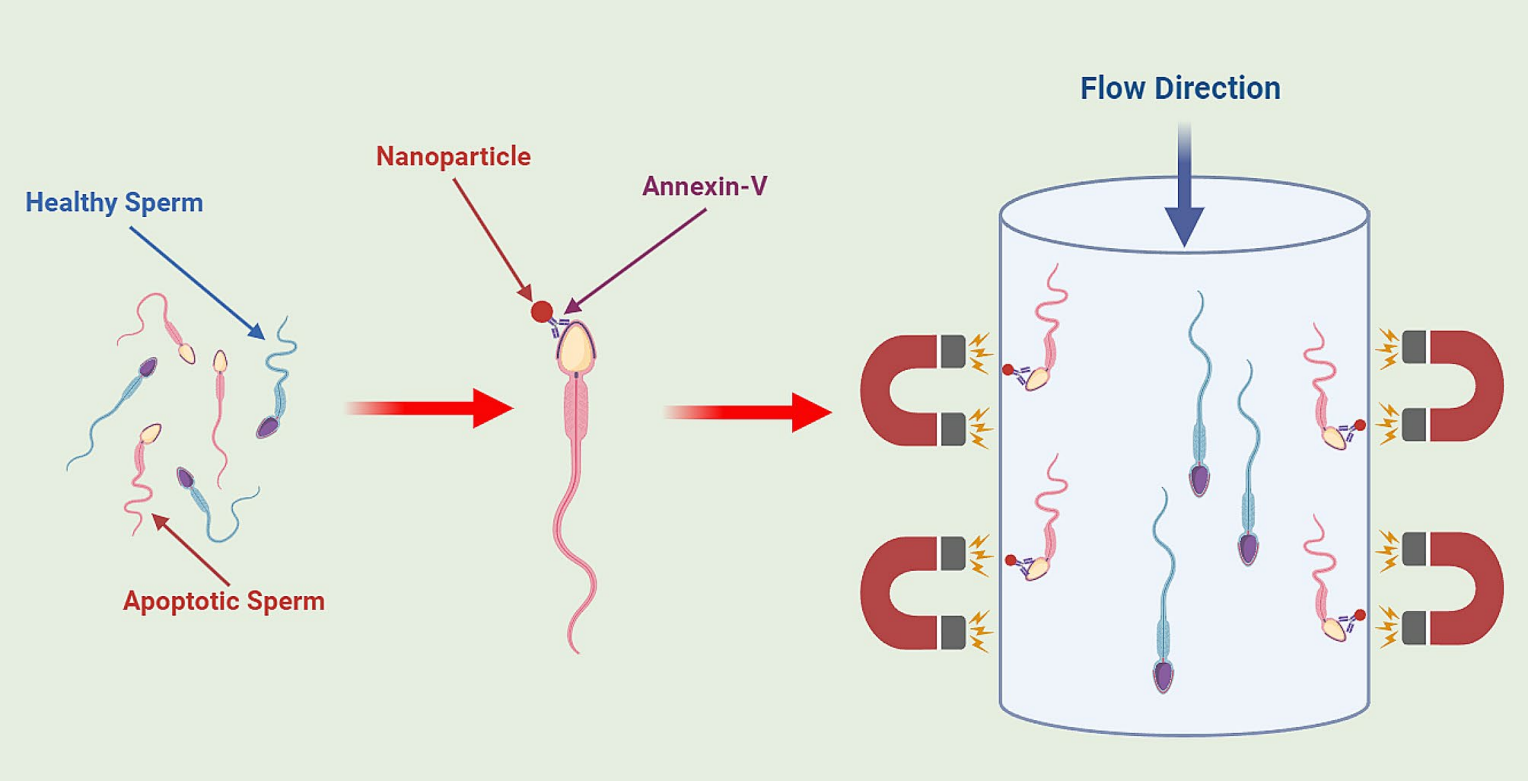
Magnetic-Activated Cell Sorting (MACS): A Visual Overview

Magnetic-Activated Cell Sorting (MACS) has emerged as a leading technology with significant potential in sperm sorting for ART. This technology functions by utilizing magnetic microbeads coated with specific antibodies to isolate target cells based on their surface markers. In the context of sperm selection, Annexin-V, a calcium-dependent phospholipid-binding protein, is frequently used as the specific antibody to identify apoptotic spermatozoa [4–7, 20–24]. Annexin-V binds to phosphatidylserine (PS), a phospholipid typically confined to the inner leaflet of the plasma membrane in viable cells(Fig. 2). Nonetheless, during the process of apoptosis, PS is externalized to the outer leaflet of the plasma membrane, thus allowing its detection and binding by Annexin-V. Comprehensive research on this technique has yielded valuable insights into its effectiveness and potential clinical applications across varied patient demographics(statistics that describe populations and their characteristics).

The employment of MACS in sperm preparation for ART offers notable advantages, including the selective isolation of viable sperm characterized by reduced DNA fragmentation and enhanced genetic integrity. Such attributes have the potential to augment both fertilization and pregnancy rates (Table 1). Studies have demonstrated the efficiency of MACS in ameliorating sperm parameters, ranging from motility and morphology to chromatin integrity, not only in normozoospermic patients but also in those presenting with suboptimal semen parameters [7, 22, 24]. The synergistic use of MACS with density gradient centrifugation (DGC) has shown particularly promising results, leading to a substantial decline in apoptotic spermatozoa and an increase in sperm quality [6, 22, 24]. Furthermore, the integration of DGC-MACS has been associated with superior ART cycle parameters, encompassing diminished sperm DNA fragmentation rates and curtailed oxidative stress, potentially leading to increased success rates in Intracytoplasmic Sperm Injection (ICSI) cycles [20].

However, certain limitations should be acknowledged (Table 1). While MACS has demonstrated encouraging outcomes within specific patient groups, its integration into ART remains nascent. Further research is required to corroborate its efficacy and to buttress the evidence advocating its inclusion in routine sperm selection protocols [20]. Additionally, the technical intricacy inherent to MACS systems could pose obstacles to its ubiquitous acceptance in clinical environments. Such technical complexity of MACS systems can be attributed to the intricate process involved in isolation of target cells based on their surface markers, utilizing magnetic microbeads and designated antibodies. This protocol mandates exacting calibration and refinement to guarantee precise and efficacious spermatozoa sorting. Moreover, the apparatus and reagents used in MACS procedures necessitate rigorous maintenance and quality assurance, amplifying the overall intricacy of assimilating this technology within clinical laboratories. Consequently, dedicated training and proficiency became indispensable for laboratory personnel to adeptly conduct MACS, potentially constraining its pervasive acceptance in routine clinical practices.

The utilization of MACS in sperm preparation for ART presents a promising approach to augment sperm quality and thereby ameliorate fertility outcomes across a spectrum of patient populations. When amalgamated with DGC, MACS has evinced notable efficacy in the exclusion of apoptotic spermatozoa, favoring the selection of viable sperm characterized by reduced DNA fragmentation, which subsequently bolsters the probability of successful fertilization and elevated pregnancy rates. Such pioneering revelations fortify the expanding corpus of evidence advocating the integration of MACS in tailored sperm selection techniques addressing male factor infertility challenges. An exhaustive appraisal of sperm quality, undertaken via various assays, inclusive of evaluations pertaining to viability, motility, chromatin integrity, and the acrosome reaction, offers an encompassing perspective on sperm functionality and genetic integrity. This rigorous analysis is pivotal for precision-driven sperm selection and the ensuing success of ART, more so in cases marked by abnormal semen parameters or idiopathic infertility. The incorporation of MACS under such circumstances underscores its potential in revolutionizing fertility treatment strategies, thereby offering hope to couples facing challenges in conception.

With the relentless progression of reproductive medicine, further research and validation studies are warranted to not only solidify these previous findings but also to probe the potential expended applications of MACS in ART. An augmented, multi-centric study encompassing a broad spectrum of patient cohorts is pivotal to bolster the empirical evidence supporting MACS’ efficacy, thereby facilitating its integration into routine sperm selection protocols in clinical settings. The adoption of innovative approaches like MACS in ART has the potential to transform the landscape of fertility treatment, exemplifying personalized care and amplifying reproductive success for a on a global scale.

### Electrophoretic sperm selection

Electrophoretic sperm selection emerges as a revolutionary modality within the realm of assisted reproductive technologies, capitalizing on the inherent electrical attributes of spermatozoa to segregate functionally adept cells [8, 25]. This technique exploits the distinctive electrical charge acquired by sperm throughout their maturation process - a characteristic that is instrumental in preventing aggregation, circumventing nonspecific binding, and mitigating undesired storage within the female reproductive tract [8]. Over time, considerable progress has been achieved in this field, leading to the refinement of methodologies that aim to ensure sample integrity, reduce DNA damage, and augment overall fertility potential [13, 23, 24]. This section delves into the advantages, limitations, addressed concerns, and future prospects of electrophoretic sperm selection.

The advent of electrophoretic sperm selection heralds a series of noteworthy advantages in the realm of assisted reproduction (Table 1). The approach introduces a non-invasive and rapid means of isolating spermatozoa with optimal fertilization potential. The CS-10 device pioneered this technique by capitalizing on the negatively charged attribute of mature sperm, resulting in the birth of viable offspring devoid of embryonic development problems [13]. The advancement embodied by the Felix™ apparatus further accentuates this advantage by incorporating a filtration system that effectively segregates contaminating cells. This results in the heightened purity and superior quality of isolated sperm populations [23]. Nevertheless, like any technological advancement, electrophoretic sperm selection presents certain limitations (Table 1). A primary challenge resides in maintaining a nuanced equilibrium between sperm membrane charge and functional attributes. Although the exclusion of negatively charged sperm through DGC may enhance DNA damage levels, the intricate dynamics of this process warrant further exploration. Moreover, the formulation of an optimized electrophoretic buffer should be a focal consideration during isolation procedure [14]. The composition and properties of the buffer critically influence the migration of spermatozoa, dictated by their charge, size, and various other determinants. Suboptimal buffer conditions could potentially lead to inconsistent results and compromised sperm quality during the separation process. The conductivity, pH, and ionic strength of the buffer necessitate rigorous optimization to ensure the precise and effective migration of sperm cells. Moreover, the constituents of the buffer should be carefully selected to mitigate potential detrimental impacts on sperm viability and function. Consequently, a comprehensive investigation of buffer formulations is essential to address this potential limitation, ensuring enhanced consistency, reliability, and reproducibility.

Additionally, the robustness of the technique across a diverse spectrum of semen samples, particularly from pathological donors, requires validation [24]. Thus, while electrophoretic sperm selection offers considerable potential, it is crucial to address these limitations to ascertain its clinical applicability.

The evolution of electrophoretic sperm selection epitomizes the dynamic nature of scientific progress in addressing pertinent concerns. The CS-10 device introduced the concept of charge-based sperm selection, revolutionizing the field and laying the foundation for subsequent advancements [13]. The Felix™ device, with its amalgamation of electrophoretic separation and a sophisticated filtration system, stands as a testament to the continual refinement of the technique. This innovation notably addresses the pivotal issue of sample purity by effectively eliminating contaminating cells [23]. Additionally, Simon et al. (2016) deepened our understanding of sperm membrane charge dynamics, highlighting the intricate relationship between charge and DNA integrity [26]. Furthermore, Ainsworth et al.‘s (2005) electrophoretic system presents a novel approach to sperm isolation, demonstrating its potential to mitigate DNA damage and enhance functional attributes [27]. The collective progress made in addressing these concerns underscores the trajectory of electrophoretic sperm selection.

The trajectory of advancements in electrophoretic sperm selection has been significant, showing great potential for the field of assisted reproduction. The transition from CS-10 to the Felix™ device exemplifies a proactive approach to addressing challenges and enhancing the efficacy of the technique [13, 23]. The studies by Simon et al. (2016) and Ainsworth et al. (2005) offer a comprehensive insight into the complex dynamics of sperm membrane charge and its implications for fertility potential [26, 27]. Additionally, these studies emphasize the importance of rigorous validation across a variety of semen samples to confirm the clinical applicability of this technique. The development of electrophoretic sperm selection is noteworthy, highlighting the capability of innovative approaches to redefine existing paradigms. As progress ensues, the field stands on the cusp of revolutionizing assisted reproduction by leveraging the electrical attributes of sperm for enhanced selection. Nevertheless, the path forward necessitates meticulous validation and comprehensive research to ensure that these advancements lead to discernible enhancements in clinical outcomes.

In the field of assisted reproductive technologies, the innovative approach of electrophoretic sperm selection presents a promising avenue for improving fertility outcomes. This technique capitalizes on the inherent electrical properties of spermatozoa, leveraging the distinct charge they acquire during maturation to facilitate selective isolation. The progression of this technique from its preliminary stages to the introduction of sophisticated devices like the Felix™ exemplifies the commitment of the field to addressing concerns and refining methodologies. Electrophoretic sperm selection offers a range of advantages, from rapid and non-invasive sperm isolation to augmented sample purity and heightened DNA integrity. Both the CS-10 and Felix™ devices, supplemented by additional pertinent studies, underscore the potential of this technique to revolutionize sperm selection, culminating in elevated success rates for assisted reproduction. However, as is the case with any emergent technology, certain challenges must be meticulously addressed. The nuanced balance between sperm membrane charge and functional attributes, coupled with the imperative for comprehensive validation across diverse semen samples, accentuates the intricacy of this technique. While electrophoretic sperm selection offers considerable potential, ongoing research and meticulous refinement are essential to guarantee its successful integration into clinical practice. In conclusion, the path of electrophoretic sperm selection is a continuous and evolving technology stream, remaining subject to further advancement and exploration. The concerted efforts of both researchers and clinicians have yielded significant progress in addressing concerns and unlocking the potential of this technique. As the field progresses, it is imperative to embrace these advancements while concurrently ensuring rigorous validation and the pursuit of improved fertility outcomes.

### Intracytoplasmic morphologically selected sperm injection (IMSI)

Intracytoplasmic Morphologically Selected Sperm Injection (IMSI) stands as a pioneering technique in assisted reproduction, with the primary objective of optimizing the selection of high-quality sperm for fertilization [10]. This advanced procedure employs high-magnification microscopy, typically around 6000x magnification, to meticulously assess sperm morphology. Through this rigorous assessment of parameters such as nuclear vacuoles, acrosomal integrity, and overall structure characteristics, IMSI seeks to pinpoint sperm that possess the optimal genetic and structural attributes [10, 28].

This approach offers a range of potential advantages [9, 11, 28](Table 1). A foremost advantage of IMSI lies in its capacity to enhance the selection of morphologically normal sperm. By leveraging higher magnification, even minute abnormalities become detectable abnormalities that might be overlooked at lower magnifications [28]. This enhanced precision is instrumental in singling out sperm with optimal genetic integrity. Moreover, the meticulous selection process of IMSI could mitigate the likelihood of transmitting genetic abnormalities or DNA damage to the developing embryo, potentially leading to improved pregnancy rates [11, 28].

However, despite its potential benefits, IMSI presents certain limitations. The exhaustive examination procedure requires considerable time, which could extend treatment durations and elevate potential patient stress [28]. Furthermore, while promising, compelling evidence delineating clear and significant improvements in clinical outcomes compared to conventional methods like ICSI remains somewhat scarce [12, 28]. Additionally, the requisites for specialized equipment and the expertise essential for high-magnification microscopy could contribute to increased costs and intricate procedures, potentially restricting accessibility for certain patients [12, 28, 29]. Firstly, the acquisition and maintenance of such advanced microscopy systems can significantly increase the overall financial strain associated with assisted reproductive treatments. The initial acquisition costs, in tandem with expenses related to routine maintenance, calibration, and prospective upgrades, amplify the financial implications of IMSI procedures compared to conventional methods. These elevated financial requirements could pose challenges for patients with limited financial means, potentially restricting their access to this advanced technique. Furthermore, the complexities associated with high-magnification microscopy necessitate an advanced degree of technical proficiency and specialized training for embryologists and laboratory personnel [29]. To attain accurate and consistent results, an in-depth grasp of the equipment is imperative, along with meticulous sample preparation and adapt interpretation of the complex morphological details shown by the high-magnification imagery. As a result, clinics offering IMSI must allocate resources towards training programs and continuous professional development for their staff members. This emphasis on specialized training and expertise adds an additional layer of complexity to the overall procedure, potentially necessitating an extended learning curve for embryologists transitioning to this technique. The combination of increased financial expenditures and heightened technical demands collectively contributes to the procedural complexity of IMSI. Although the prospective advantages of IMSI are substantial, the requisites for specialized apparatus, continual maintenance, and advanced technical expertise might render its adoption more challenging for certain clinics and patients. As the field of assisted reproduction endeavors towards inclusivity and egalitarian access to advanced treatments, devising strategies to surmount these challenges emerges as a critical priority. Efforts to enhance training programs, explore cost-effective equipment alternatives, and foster collaborations among clinics could pave the way toward broader accessibility of IMSI. This would ensure that patients from various socio-economic backgrounds have the opportunity to benefit from this advanced sperm selection technique [12, 28, 29].

The subjective element intrinsic to embryologists’ assessment of sperm morphology during the selection process carries the risk of introducing selection bias and result in variability of outcomes [12, 28]. This significant concern emphasizes the necessity for standardized criteria and comprehensive methodologies in sperm selection techniques, such as IMSI. Any deviations can markedly impact the success rates and overall efficiency of assisted reproductive procedures. Efforts toward standardization encompass the establishment of comprehensive guidelines for sperm selection criteria and morphological assessments in IMSI. The primary objective is to reduce observer variability. Ongoing work is carefully creating consistent rules to accurately define the parameters for selecting sperm and to enhance the accuracy of morphological assessment within the IMSI process [28]. This systematic approach aims to effectively diminish the impact of observer variability, ensuring a consistent and objective methodology for embryologists in the process of sperm selection. By minimizing inherent subjectivity through these guidelines, the overall reliability of IMSI outcomes could be markedly augmented, consequently translating into elevated success rates for assisted reproductive procedures. Furthermore, instituting standardized criteria might facilitate the development of exhaustive training initiatives for embryologists, ensuring their proficiency in accurately identifying and selecting morphologically normal sperm. This pedagogical aspect is crucial in ensuring uniformity and competence across different clinical settings. Furthermore, the adoption of standardized guidelines might foster collaboration within the reproductive medicine community, facilitating the exchange of best practices and continuous refinement of the IMSI technique.

As the path to standardization continues, it is imperative that these efforts remain adaptive to new research findings and technological advancements. By directly confronting the issue of observer variability, the field of assisted reproduction may advance towards elevated precision, ultimately benefiting couples seeking fertility treatments. Further exploration delves into the long-term health and developmental outcomes of embryos derived from IMSI-selected sperm [9, 28]. Researchers are actively exploring the combined use of IMSI with other advanced sperm selection techniques to leverage their potential synergistic benefits [11, 12, 28]. Moreover, recent studies have investigated integrating techniques like motile sperm organelle morphology examination (MSOME) with IMSI to address cases of unexplained infertility [11].

In conclusion, IMSI holds substantial promise within the domain of assisted reproduction [10]. Through the utilization of high-magnification microscopy and stringent morphological criteria, IMSI offers the potential to elevate the quality of selected sperm, ultimately contributing to improved embryo quality and heightened pregnancy rates [9–11, 28]. Nonetheless, the present evidence underscores the imperative for ongoing research, efforts towards standardization, and a holistic exanimation to thoroughly assess the efficacy of the technique and its implications for assisted reproductive practices [9, 11, 12, 28, 29].

**Table 1:** Advantages and limitations of various sperm selection techniques

	Microfluidics	MACS	Electrophoretic Sperm Selection	Intracytoplasmic Morphologically Selected Sperm Injection (IMSI)
**Principles**	- Utilizes advanced principles for isolating spermatozoa based on functionality and morphology [1–3].	- Utilizes magnetic microbeads coated with specific antibodies, such as Annexin-V, to isolate target cells based on their surface markers [4–6, 20–24].	- Leverages spermatozoa’s electrical properties for selective isolation [8, 25].	- High-magnification microscopy used to select sperm with optimal genetic and structural attributes [10, 28].
**Selectivity and Specificity**	- Offers high selectivity for motile and morphologically normal sperm, improving selection outcomes [4, 5].	- Selectively isolates viable sperm with reduced DNA fragmentation, enhancing fertilization and pregnancy rates [7, 22, 24].	- A rapid, non-invasive isolation technique ensures high purity and quality [13, 23].	- Enhances the selection of morphologically normal sperm, improving genetic integrity and pregnancy rates [9, 11, 28].
**Cost-Effectiveness**	- Initial costs are higher due to specialized equipment, but benefits in outcome efficiency may offset these costs [6, 7].	- Integration costs and technical complexity need further research to justify widespread clinical use [20].	- Requires further validation; optimization of buffer formulations is needed [14].	- Specialized equipment and advanced expertise increase costs and procedural complexity [12, 28, 29].
**Efficiency**	- Enables real-time monitoring and analysis, increasing ART outcome efficiencies [8, 9].	- Combined with density gradient centrifugation, it shows improved sperm quality and ART success [6, 22, 24].	- Promising in reducing DNA damage and enhancing sperm quality [8, 23, 25].	- Potential to reduce genetic abnormalities and DNA damage in embryos [11, 28].
**Standardization**	- Crucial to standardize device fabrication and protocols; variations may occur across labs [10, 11].	- High technical complexity; requires dedicated training for lab personnel [20].	- Essential for consistent results across different samples and settings [14, 24].	- Aims to reduce observer variability in sperm selection and morphological assessments [12, 28].
**The amount of sperm samples required**	- Requires minimal semen, beneficial for cases with limited sample availability [12, 13].	- Efficiently selects viable sperm, minimizing the need for large samples [20].	- Efficiently isolates viable sperm, reducing the need for large samples [8, 25].	- Requires meticulous sample preparation; extended treatment durations may increase patient stress [28].
**Ease of Implementation**	- Requires specialized technical expertise and training [14–17].	- Meticulous calibration and quality assurance are needed for clinical integration [20].	- Optimization of buffer compositions and device operation necessary [14, 24].	- High technical proficiency and extended learning curve required for embryologists [29].

### Advanced sperm quality assessment technology

The continuous advancement of technology has unveiled new frontiers in the field of male fertility assessment, offering more sophisticated methods to evaluate sperm quality for successful assisted reproduction [30]. While traditional semen analysis has long been the cornerstone of assessing male fertility, the limitations of this approach have driven the development of advanced techniques that provide a deeper understanding of sperm characteristics [31]. These innovative methods offer the potential to revolutionize how we assess and address male infertility, shedding light on previously unexplored aspects of sperm quality [13].

#### Sperm chromatin structure assay (SCSA)

The Sperm Chromatin Structure Assay (SCSA) is a technique that evaluates DNA fragmentation in sperm, a critical factor influencing fertilization and subsequent embryo development. This method engages acridine orange staining in conjunction with flow cytometry to ascertain the susceptibility of sperm DNA to denaturation [31]. Given the intimate correlation between the integrity of sperm DNA and reproductive success, the SCSA emerges as an indispensable instrument in the evaluation of male fertility [32].

SCSA provides a comprehensive assessment of DNA fragmentation in sperm, contributing to an enhanced comprehension of prospective reproductive results. By quantifying the degree of DNA denaturation, SCSA offers insights into the sperm’s ability to support successful fertilization and embryo development. However, the intricate nature of SCSA necessitates the use of specialized equipment and expertise, potentially heightening the intricacy and expense associated with assessments. Additionally, although SCSA proffers crucial insights into DNA fragmentation, establishing a direct correlation with clinical outcomes remains inconsistent, underlining the need for additional research [31].

#### Zeta potential analysis

Zeta potential is an indicator of the surface charge of sperm cells. This analysis is based on the principle of electrophoresis, which measures the movement of charged particles in an electric field, it offers insights into sperm capacitation and their interaction with the female reproductive tract [14]. As such, a deeper comprehension of the zeta potential and its role in sperm behavior can be pivotal for predicting fertilization success [33].

Zeta potential analysis provides a unique perspective on sperm functionality by assessing surface charge dynamics. The evaluation of these dynamics can offer insights into sperm capacitation and its role in successful fertilization. While Zeta potential analysis holds promise, its translation into clinical practice might require overcoming challenges related to standardization and establishing clear reference values. Additionally, the complex interplay of a range of factors affecting zeta potential dynamics in vivo necessitates further research [14].

The integration of advanced sperm quality assessment methodologies marks a monumental advancement forward in the field of assisted reproduction. This potential renaissance stands poised to reshape the landscape of reproductive medicine. The nexus of technological innovation with the intricacies of reproductive biology may serve as the catalyst for optimized outcomes for couples grappling with infertility challenges [13]. While the path to progress is undeniably accompanied by inherent challenges, it is the persistent commitment to exhaustive research, rigorous standardization, and seamless clinical implementation that will bridge the gap between these cutting-edge techniques and their transformative potential. This journey, powered by the formidable capabilities of technology, is set to illuminate and enrich the path toward parenthood.

As these advanced methods gain traction, the imperative need for standardized protocols and benchmark reference values becomes evident [13, 30, 32, 34]. Through their integration into routine clinical practice, these methods hold the potential to substantially enhance the outcomes of assisted reproduction, granting clinicians with insightful tools for judicious treatment decisions [33]. By delving deeper into various sperm parameters, these methodologies offer a comprehensive evaluation of male fertility potential. Despite the complexities inherent to innovation, ongoing research endeavors and the pursuit of standardization will be the linchpin in realizing the full transformative scope of these advanced techniques [13, 30–32, 34].

In this era of reproductive medicine, the integration of technological advancements with sophisticated medical insight facilitates progress towards enhanced and individualized assisted reproduction outcomes. Navigating this evolving terrain, the potential of advanced sperm quality assessment techniques emerges as a pivotal factor, expanding the horizons of parenthood and offering hope to those pursuing its rewards.

### Comparison of conventional and advanced sperm selection methods

#### Evolution of sperm selection techniques

Advancements in sperm selection techniques have been profound, catalyzed by the unyielding endeavor to enhance outcomes within ART [35]. The evolution of sperm selection methodologies can be delineated into three pivotal stages: the establishment of historical foundations, the refinement of conventional techniques through iterative development, and the recent integration of sophisticated approaches. In the initial stages, traditional sperm selection techniques, namely swim-up and DGC, established the foundational protocols that catalyzed further advancements in the field [15, 36]. These methodologies, which have been fundamental in ART clinics for many years, function by isolating motile spermatozoa through their intrinsic motility or by employing density gradient centrifugation to segregate spermatozoa based on their specific density. These techniques formed the bedrock for preparing high-quality sperm for fertility procedures. As time progressed, enhancements in these traditional methodologies have become manifest. Decades of empirical research and clinical experience have been instrumental in refining the swim-up and DGC techniques [37]. They have evolved into reliable tools for the efficient isolation of motile sperm and elimination of contaminants, ultimately enhancing the chances of successful fertilization.

A range of innovative methods for sperm selection has emerged over the past few years and heralded the advent of a new era characterized by advanced technologies [16, 38, 39]. These methods leverage on cutting-edge technologies to target distinct attributes of sperm quality. IMSI employs high-magnification microscopy to select sperm based on morphological criteria, whereas microfluidic sperm sorting utilizes microscale channels and fluid dynamics to augment the precision of selection. Zeta potential-based selection utilizes the electrical charge on sperm surfaces, whereas sperm chromatin dispersion (SCD) measurements specifically target DNA fragmentation assessment. This advanced technology exemplifies the field’s commitment to addressing complex facets of sperm quality through customized solutions.

In summary, the trajectory of sperm selection methodologies illustrates a continual progression from foundational practices to refined conventional techniques, culminating in the introduction of advanced methods. This progression underscores the unwavering dedication to enhancing fertility treatment outcomes and demonstrates the dynamic nature of reproductive medicine. Each stage of development enriches the framework for a more comprehensive and personalized approach to sperm selection, congruent with the paramount objective of optimizing ART success.

#### Conventional vs. advanced sperm selection techniques: a comparative analysis

There exists a between traditional and innovative methods in the field of sperm selection. This section presents a thorough comparative analysis of these approaches, shedding light on the core differences in their underlying principles, distinct focal points on physical and functional attributes, the crucial role of technology in shaping advanced techniques, the utilization of specialized tools and materials, and the future implications that accompany the shift from conventional to advanced methodologies.

At its core, the distinction between conventional and advanced techniques lies in their foundational principles. Traditional methods, typified by DGC and swim-up approaches, are predicated on sedimentation principles to isolate sperm with desirable physical characteristics [16, 28, 35–37]. In contrast, advanced techniques, as demonstrated by IMSI and Zeta potential-based selection, are grounded in more sophisticated methodologies that delve into the complex interactions of spermatozoa’s functional attributes, such as DNA integrity and gene expression [4, 21, 23, 25, 31].

The divergent emphases of these techniques are markedly distinct. Conventional methods focus primarily on physical traits, such as motility and morphology, as indicators of sperm quality [16, 28, 35–37]. In contrast, advanced methods, exemplified by IMSI and Zeta potential-based selection, concentrate on functional attributes as potent determinants of fertilization success [29, 34]. This shift represents a transformative progression, acknowledging that the genetic integrity and molecular characteristics of sperm are fundamentally interconnected with their ability to achieve successful fertilization.

Technology serves as a pivotal catalyst in the evolution of advanced sperm selection techniques. Microfluidic platforms, representing the forefront of innovation, utilizing fluid dynamics to meticulously manipulate and segregate sperm based on their attributes [9, 28]. Such technological advancements permit the rapid and precise selection of sperm with optimal qualities, thereby revolutionizing the paradigm of sperm selection methodologies. Utilizing specialized tools and materials further underscores the chasm between conventional and advanced approaches. Microfluidic devices, for instance, employ intricate channel designs to facilitate controlled sperm separation, enabling improved DNA integrity and motility [9, 28]. Similarly, Zeta potential-based selection leverages unique chamber configurations to exploit sperm’s intrinsic charge, exemplifying the ingenuity inherent in advanced methods [34].

Technology functions as a pivotal driving force in the evolution of advanced reproductive selection techniques. Microfluidic platforms have risen to prominence as state-of-the-art instruments, utilizing fluid dynamics to meticulously control and categorize spermatozoa according to their distinct characteristics [9, 28]. Such technological advancements facilitate the swift and accurate identification of spermatozoa possessing desirable qualities, thereby transforming the field of sperm selection methodologies. The utilization of specialized tools and materials distinctly emphasizes the chasm between conventional and advanced approaches. For example, microfluidic devices utilize complex channel designs to enable precise sperm separation, thereby enhancing DNA integrity and motility [9, 28]. Similarly, Zeta potential-based selection leverages specialized chamber configurations to tap into the inherent electrical charge of spermatozoa, showcasing the innovation embedded in advanced methods [34].

The transition from conventional to advanced methods holds profound future implications. Clinical trials have showcased the potential of these advanced techniques to yield superior outcomes. Zeta potential-based selection has been shown to improve embryo quality and increase the rate of successful pregnancies compared to traditional DGC methods [34]. Likewise, microfluidic sperm sorting has demonstrated the capability to enhance sperm concentration, motility, and embryo quality in ICSI treatments [9]. This empirical evidence indicates a paradigm shift, wherein functional attributes are increasingly recognized as paramount in maximizing treatment success.

The comparative analysis of conventional and advanced sperm selection techniques encompasses multiple dimensions (Table 2). Spanning from foundational principles to technological advancements, physical attributes, and functional intricacies, this analysis underscores the profound shift in the approach to treating male factor infertility. As advanced methodologies increasingly gain prominence, the synergistic interplay of science and technology ushers in new frontiers, heralding more precise, efficacious, and personalized approaches to enhancing fertility.

**Table 2:** Comprehensive Comparison of Conventional and Advanced Sperm Selection Techniques

Aspect	Conventional Techniques	Advanced Techniques
**Underlying Principles**	- Rely on sedimentation principles using centrifugation.	- Rooted in sophisticated methodologies exploring functional attributes.
	- Isolate sperm based on their natural swimming ability.	- Focus on sperm’s DNA integrity, gene expression, and capacitation [1–3, 40–42].
	- Prioritize physical traits such as motility and morphology.	- Emphasize the critical role of DNA integrity and molecular attributes.
**Technological Innovation**	- Relatively limited use of advanced technology.	- Leverage microfluidic platforms and cutting-edge imaging techniques [4, 40, 43, 44].
	- Traditional centrifuges used for separation.	- Utilize microscale channels, unique tools, and molecular analysis.
**Utilized Tools and Materials**	- Use traditional centrifuges, density gradients for separation.	- Employ microfluidic devices, specialized chambers for sorting [5, 40–42].
	- Basic materials like tubes, density gradient media.	- Employ advanced molecular analysis instruments for assessment.
**Functional Assessment**	- Limited precision in selecting specific sperm.	- Achieve high precision in selecting sperm based on attributes.
	- Focus on basic motility and morphology assessment.	- Evaluate DNA integrity, capacitation potential, and gene expression [6, 45–47].
	- Limited assessment of functional attributes beyond motility.	- Comprehensive evaluation of sperm’s molecular properties.
**Outcome Predictability**	- Limited prediction of fertilization success based on morphology.	- Enhanced prediction based on functional attributes and DNA integrity [7, 48–51].
**Clinical Applicability**	- Success rates primarily linked to morphology-based selection.	- Improved embryo quality, higher chances of successful pregnancies [8, 52–55].
	- Limited potential for targeted selection based on functional traits.	- Potential cost-effectiveness, reduced treatment duration for patients.
	- Established in ART clinics for years, regarded as standard procedures.	- Shaping the future of assisted reproduction with revolutionary methods.

The transition from traditional to improved sperm selection approaches constitutes a substantial advancement in the field of ART. Conventional approaches, which rely on sedimentation and density principles, have historically formed cornerstone of sperm selection in ART. These conventional methods predominantly focused on physical characteristics, such as motility and morphology. While these factors are undoubtedly important, they offer only a limited insight of the complicated world of male fertility. However, the advent of advanced techniques marks a fundamental shift in this field. These methods, grounded in the foundations of sophisticated scientific methodologies and cutting-edge technology, rigorously investigate the functional attributes of sperm. These methods take into account factors such as DNA integrity, gene expression, and capacitation potential, acknowledging the critical role these molecular attributes play in the path to successful fertilization. This transition underscores the significance of comprehending sperm at a molecular level, transcending the focus on mere physical characteristics.

The empirical evidence underpinning this shift is compelling. Clinical trials have consistently demonstrated the capability of advanced techniques to surpass the performance of their conventional counterparts. For instance, Zeta potential-based selection has demonstrated notable efficacy, improving embryo quality and increasing the likelihood of successful pregnancies, particularly when compared to traditional DGC methods [15, 34, 35]. Similarly, microfluidic sperm sorting has demonstrated its effectiveness by elevating sperm concentration, motility, and embryo quality in ICSI treatments [4, 56]. These findings signify a paradigm shift where functional attributes increasingly assume a central role in optimizing treatment success.

This comparative analysis between conventional and advanced sperm selection techniques encompasses multiple dimensions (Table 2). It represents not merely a transition in methodology but signifies the evolution of an entire field. The synergistic interplay of science and technology is forging new frontiers, heralding the advent of more precise, effective, and personalized approaches to enhancing fertility. Advanced methodologies transcend mere techniques; they epitomize a transformative journey toward more successful ART outcomes and actualizing the aspirations of parenthood.

In conclusion, the transition from conventional to advanced sperm selection techniques exemplifies the extraordinary potential of medical science and innovation to transform and expand the realm of possibilities. This advancement offers newfound hope to individuals and couples struggling with infertility challenges. As advanced techniques increasingly gain prominence, the future holds the promise of more tailored, efficient, and effective solutions, ultimately enhancing the probabilities of successful pregnancies and fulfilling the fundamental human aspiration for parenthood [9, 29, 39].

#### Future directions and implications

The evolution of sperm selection techniques from conventional methods to advanced approaches has revolutionized assisted reproduction, opening avenues for a future replete with possibilities. This section examines the transformative impact of advanced sperm selection methods and delves into the future implications of these developments. A comprehensive table detailing the evolution of sperm selection techniques, their benefits for commercialization in the ART industry, and their congruence with patient preferences is included (Table 3). This table provides an overview of the strategic advantages of advanced methods, delineating their role as essential elements of contemporary assisted reproductive technology.

**Table 3:** Evolution and Commercialization Potential of Advanced Sperm Selection Technique

Sperm Selection Technique	Evolution from Conventional Technique	Benefits for Commercialization in ART Industry
**Microfluidic Sperm Sorting** [1–3, 40, 41, 43, 44]	Enhanced precision and real-time observation	Offers potential cost-effectiveness and reduced treatment duration.
	Overcomes limitations of motility-based selections	Attracts a broader patient base seeking advanced and efficient ART options.
	Offers real-time observation of sperm behavior	Elevates clinics’ competitive edge by providing state-of-the-art techniques.
	Allows sorting based on functional attributes beyond motility	Meets the growing demand for improved ART success rates and patient satisfaction.
**Magnetic-Activated Cell Sorting (MACS)** [4–6, 45–47, 57]	Offers efficient and gentle sorting, minimizing potential damage to sperm	Aligns with patient preferences for minimally invasive procedures.
	Shifts from physical manipulation to targeted functional selection	Enhances clinics’ reputation as leaders in advanced reproductive technologies.
	Enables rapid and gentle sorting without mechanical stress	Meets the demand for improved ART success rates and patient satisfaction.
	Offers the ability to select sperm based on specific criteria such as DNA integrity	Enhances clinics’ reputation as leaders in advanced reproductive technologies.
**Intracytoplasmic Morphologically Selected Sperm Injection (IMSI)** [9–11, 48–51]	Raises the standard of sperm selection, attracting patients seeking premium treatments	Addresses the increasing demand for enhanced embryo quality and success rates.
	Transforms selection from basic motility to sophisticated morphological evaluation	Expands clinics’ market share by catering to those seeking top-tier fertility solutions.
	Offers enhanced precision in choosing sperm with optimal morphology	Provides potential for improved fertilization outcomes by selecting sperm with better structural integrity.
**Zeta Potential Analysis** [12, 14, 52, 53]	Aligns with the trend toward holistic assessments for comprehensive fertility solutions	Positions clinics as pioneers in holistic reproductive medicine.
	Expands assessment beyond basic attributes to evaluate sperm functional properties	Establishes clinics as go-to centers for addressing complex infertility cases.
	Offers insights into capacitation, a critical step for successful fertilization	Attracts patients looking for innovative technologies that promise higher success rates.
**Sperm Chromatin Structure Assay (SCSA)** [13, 15, 54, 58, 59]	Addresses the growing awareness of the impact of DNA integrity on fertility outcomes	Meets the demand for comprehensive fertility evaluations with a focus on genetic integrity.
	Transcends traditional assessments by focusing on DNA integrity	Attracts patients seeking advanced assessments to maximize their chances of conception.
	Offers a deeper understanding of a crucial factor affecting reproductive success	Establishes clinics as leaders in addressing DNA-related fertility challenges.

The groundbreaking advancements in sperm selection have heralded the advent of a new era in assisted reproduction. Transitioning from basic motility assessments, the field has evolved to sophisticated functional and genetic evaluations, yielding enhancements in fertilization rates, embryo quality, and pregnancy outcomes [39, 56]. As these techniques increasingly gain prominence, there emerges a need to reconcile the advantages they provide with the proven historical success of conventional methods that have withstood the test of time [16, 36]. Customization and individualized approaches are increasingly recognized as crucial factors in determining the most appropriate sperm selection method for each specific case. Patient-centric care is now enhanced by the availability of diverse techniques, enabling fertility specialists to customize treatments in accordance with individual needs and preferences [15, 34]. The integration of advanced sperm selection methods empowers clinicians to precisely address complex cases, thereby offering patients an increased likelihood of successful outcomes [39, 56].

In the realm of assisted reproduction, the significance of ongoing research and development is paramount. The current advancements represent merely a stepping stone towards a future wherein sperm selection outcomes are optimized to an unprecedented level. Scientific inquiry dedicated to refining existing techniques and exploring novel methodologies, as exemplified in reference [4], will persistently influence landscape of fertility treatments [37, 38]. The synergistic interplay of clinical insights and technological innovation hold the promise of elevating the success rate of assisted reproduction.

The incorporation of advanced technologies into assisted reproductive practices engenders a sense of optimism for the future advancements. As these methods grow more accessible and sophisticated, the spectrum of fertility treatments expended. Both patients and clinicians can look forward to more personalized, efficacious, and streamlined solutions, potentially resulting in increased rates of successful pregnancies and the realization of aspirations for parenthood [29, 39]. The transition from conventional to advanced sperm selection techniques epitomizes the potential of science and innovation to reshape lives and forge new frontiers in assisted reproduction.

#### Charting progress: a holistic perspective

The evolution from conventional sperm selection methods to advanced techniques signifies a transformative journey, profoundly impacting the landscape of assisted reproduction. This section conducts a comprehensive analysis of this transformative journey, providing a holistic perspective on its implications and far-reaching impact. The transition from motility-based assessments to methods focused on functional attributes and genetic integrity, as detailed in reference [4], constitutes a defining characteristic of this transformation. Each advancement in this field has been propelled by the dual goals of achieving higher success rates and improved patient experiences [39, 56]. This evolutionary trajectory serves as a testament to the resilience of the field, exemplifying its commitment to pushing boundaries and unlocking novel avenues in fertility treatment.

As advanced sperm selection methods assume a central role, their implications extend well beyond the walls of laboratories and clinics. These techniques have elevated the likelihood of successful conception and fundamentally transformed the dialogue around infertility. These advancements have fostered optimism and enabled couples to consider a wider array of fertility treatments, each specifically tailored to their unique needs and circumstances [4, 25]. The far-reaching impact of these advancements extends beyond individual cases, potentially altering societal attitudes toward assisted reproduction. The success stories emerging from advanced techniques, including those studied in reference [4], possess the capability to inspire, educate, and encourage a broader acceptance of these technologies. As more families experience the joy of parenthood through these methods, the perception of infertility can gradually evolve from a source of stigma to one of resilience and hope [25, 56].

In conclusion, the transformative journey from conventional to advanced sperm selection methods epitomizes the union of scientific innovation and the human quest for parenthood. This evolution stands as a testament to the extraordinary ability of medical science to transcend limitations and redefine the realm of possibilities. The impact of these advancements reaches beyond merely improving pregnancy rates; it profoundly affects the lives of individuals and families. For many, the long-held dream of parenthood has been realized through the dedicated efforts of clinicians, researchers, and groundbreaking technologies.

## Conclusion

In conclusion, the transformation from traditional to advanced sperm selection techniques exemplifies the integration of scientific innovation with the pursuit of parenthood, signifying a major advancement in ART. Methods such as microfluidic sperm sorting, MACS, and IMSI each uniquely enhance gamete and embryo quality by prioritizing genetic integrity and functional competence. This progression towards more accurate and inclusive gamete selection marks a pivotal change in fertility treatments, aiming for personalized, accessible, and ethically sound options. The effects of these innovations go beyond merely increasing pregnancy rates; they profoundly impact the lives of individuals and families for whom the dream of parenthood is realized through the combined efforts of clinicians, researchers, and cutting-edge technologies. As these techniques advance, they promise to enhance success rates and overcome existing challenges, steering ART toward more equitable and effective solutions. Ultimately, these developments lay the foundation for the future of personalized fertility care, offering promising prospects for optimized outcomes and enhanced patient care, thus transforming societal views on infertility from a condition marked by stigma to one characterized by resilience and hope.

## Data Availability

No datasets were generated or analysed during the current study.

## Abbreviations

ART: Assisted Reproductive Technology
MACS: Magnetic-Activated Cell Sorting
IMSI: Intracytoplasmic Morphologically Selected Sperm Injection
PDMS: polydimethylsiloxane
PS: phosphatidylserine
DGC: density gradient centrifugation
ICSI: Intracytoplasmic Sperm Injection
MSOME: motile sperm organelle morphology examination
SCSA: Sperm Chromatin Structure Assay
SCD: sperm chromatin dispersion
